# The Treatment of Fracture of the Patella

**Published:** 1905-12-16

**Authors:** 


### THE TREATMENT OF FRACTURE OF THE PATELLA.

At a meeting of the Medico-Chirurgical Society of Edinburgh on December 6, Mr. G. G. Hamilton read a paper on the treatment of fractured patella by transverse wiring. He said that, while operations on the knee-joint require the most scrupulous asepsis, if this condition is fulfilled wiring gives the best results in the shortest time and is therefore that which should be chosen for patients in whom the limb will be subjected to severe strain in the course of their occupation. A semi-lunar incision is made with the convexity downwards, which at its lowest part reaches to the lower fragment. The joint is opened and cleared of blood and any loose fragments of bone, but the patella should not be separated from the soft parts. The two fragments are then drilled transversely and brought together by strong silver wire. The patient may be allowed to move the knee about ten days after the operation. Radiographs shown by the lantern, demonstrated that osseous union was readily secured in this way, while by other methods though fairly good functional results were obtained, there was often great separation of the fragments and bony union was extremely rare. A lad was shown who had fractured both patellæ and was treated by operation a year ago with excellent results. In another patient, who had sustained a fracture of the left patella nine years ago and wore a splint for many months, a fracture on the right side had been wired a month ago. This man stated that his right leg was as useful after a month's treatment as the left had been after eighteen months. In a patient who died of apoplexy some weeks after operation the wound had healed and the fragments of the patella were found to be firmly united.

